# Discontinuity of psychiatric care among patients with bipolar disorder in the Netherlands

**DOI:** 10.1177/00207640241278291

**Published:** 2024-09-04

**Authors:** Arnold PM van der Lee, Adriaan Hoogendoorn, Ralp Kupka, Lieuwe de Haan, Aartjan TF Beekman

**Affiliations:** 1 Department Psychiatry, Amsterdam University Medical Centre – VUmc, The Netherlands; 2 Department Psychiatry, Amsterdam University Medical Centre – AMC, The Netherlands

**Keywords:** Bipolar disorder, continuity of care, practice variation, mental health services, logistic regression

## Abstract

**Background:**

Patients with bipolar disorder benefit from guidelines recommended continuous community-oriented psychiatric and somatic healthcare, but often discontinue psychiatric care.

**Aims:**

The first objective was to identify predictive factors of discontinuity of psychiatric care among patients who had received psychiatric care. The second objective was to examine if practice variation in discontinuity of psychiatric care existed between providers of psychiatric care.

**Method:**

Registry healthcare data were used in a retrospective cohort study design using logistic regression models to examine potential predictive factors of discontinuity of care. Patient-related predictive factors were: age, sex, urbanization, and previous treatment (type and amount of psychiatric care, alcohol, and opioid treatment). Patients already diagnosed with bipolar disorder were selected if they received psychiatric care in December 2014 to January 2015. Discontinuity of psychiatric care was measured over 2016.

**Results:**

A total of 2,355 patients with bipolar disorder were included. In 12.1% discontinuity of care occurred in 2016. Discontinuity was associated with younger age and less outpatient care over 2013 to 2014. Discontinuity of patients who received all eight quarters outpatient care including BD medication was very low at 4%. The final model contained: age, type of psychiatric care, and amount of outpatient care in 2013 to 2014. Practice variation among providers appeared negligible.

**Conclusions:**

The (mental) health service in the Netherlands has few financial or other barriers toward continuity of care for patients with severe mental disorders, such as bipolar disorder. An active network of providers, aim to standardize care. This seems successful. However, 12% discontinuity per year remains problematic and more detailed data on those most at risk to drop out of treatment are necessary.

## Introduction

Bipolar disorder (BD) is a severe mental illness characterized by recurrent manic and depressive episodes separated by intervals of various durations, often beginning in adolescence or early adulthood and extending over a lifetime. Next to the burden of symptomatic episodes, it affects quality of life, psychosocial, and occupational functioning. Patients often have somatic comorbidities and a considerable shorter life expectancy (Aldinger & Schulze, 2017; Correll et al., 2015; Dols et al., 2018; Kroon et al., 2013; Kupka et al., 2015; Plans et al., 2019; Uher et al., 2019). As in other severe chronic illnesses, patients benefit from guideline recommended continuous community-oriented psychiatric and somatic healthcare integrated with community services. Patients with bipolar disorder who are stable on maintenance treatment for at least 2 years, and do not require a complex pharmacological treatment, can be referred to the general practitioner. In case of recurrence, referral back to acute specialized care must be possible. Continuous integrated healthcare treatment may prevent episode recurrence, acute interventions such as crisis treatment and hospitalization, and reduce early mortality. However, continuous integrated healthcare is not easy to achieve even in well-endowed health systems (Crawford et al., 2004; D’Avanzo et al., 2023; Hoertel et al., 2014; Kupka et al., 2015; Laan et al., 2010; Lublóy et al., 2020; O’Neill et al., 2019; Park et al., 2024; van der Lee, et al., 2021; Zyto et al., 2020). Barriers to continuous care include not only limitations in the access to sufficient trained personnel and financial restraints, but also mind-set and regulatory issues that favor short-term interventions aiming at short-term amelioration of symptoms over longer term investment in the health and functioning of patients and prevention of relapse. This is not only a problem in lower or mid-income countries, but also in countries like the Netherlands, with a well-endowed health system.

Discontinuity of guideline recommend psychiatric care among patients with BD can be attributed to patient characteristics, previous psychiatric healthcare utilization, provider characteristics (practice variation), and the organization of the healthcare system (Fischer et al., 2008; Kroon et al., 2013; Nosé et al., 2003; Peen et al., 2010, 2020). Previous psychiatric treatment can be prognostic for discontinuity of care (Crawford et al., 2004; Uher et al., 2019). Alcohol and opioid and other substance dependency often complicates treatment and this may further affect continuity of care (Salloum & Brown, 2017; Spijker et al., 2019). Practice variation between providers of care has been studied frequently, but studies of practice variation in mental healthcare are less common (Andrilla et al., 2018; D’Avanzo et al., 2023; de Ruiter et al., 2013, 2015; Edbrooke-Childs et al., 2020; Golberstein et al., 2015; Kim et al., 2017; O’Neill et al., 2019; Park et al., 2024; Vassos et al., 2016; Watson et al., 2019; White et al., 2014). Differences between providers in the quality, quantity, and outcomes of psychiatric healthcare for patients with BD can be used to identify best practices.

Our aims were (i) to test continuity of care among bipolar patients using national data, (ii) contrasting different sources of discontinuity, and (iii) with a special interest in the effects of practice variation among providers of care. Given the emphasis on avoiding financial or other barriers for patients with severe mental illness to access continuity of care, we hoped to find high levels of continuity of care. We expected to find that discontinuity of care would be multifactorial determined. Given the well-developed network of care providers in the Netherlands, we hoped to find relatively little practice variation

## Method

### Study design and patient selection

A retrospective longitudinal design was used to study discontinuity of psychiatric care. All data were derived from the health insurance registry of the Dutch health insurer Zilveren Kruis.

Inclusion criteria were: patients were diagnosed with BD in 2013 to 2014 and had received at least 1 day of psychiatric care (outpatient care with or without guideline-recommended medication for BD) from December 2014 to January 2015. Patients were insured by Zilveren Kruis for the entire study period 2013 to 2016 and were 18 to 69 years of age on 1-1-2015. Only patients allocated to providers with at least 50 patients with BD insured by Zilveren Kruis were selected. All patients insured by Zilveren Kruis who met the selection criteria were included.

**Figure 1.**
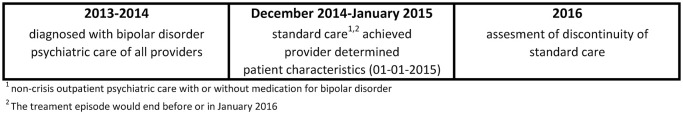
Study design.

The study design (Figure 1) proceeded as follows: (1) Inclusion: patients diagnosed with BD in 2013 to 2014 who achieved outpatient psychiatric care in December 2014 to January 2015. (2) Follow-up: over the year 2016, discontinuity of outpatient psychiatric care was assessed.

### Data source: Dutch computerized health insurance registry data

The Dutch health insurance companies are private companies but are well regulated by the government and strictly monitored by the Dutch Healthcare Authority (NZa; NZA, 2024). The Dutch Health Insurance Act requires that insurance companies administer their registries of claims and other insurance data very accurately. Zilveren Kruis is the largest health insurance company in the Netherlands and insures 30% of the population.

Specialized psychiatric and somatic healthcare is financed by the diagnosis treatment system (DBC). A psychiatric DBC-claim can continue up to 365 days. The fees for outpatient care are set per minute, for inpatient care per day and intensity of care. The amount of psychiatric care a patient received, both inpatient and outpatient care, were computed using these fees of DBC-claims. Medication for BD in the dataset is the medication which is picked up at the pharmacy.

Selection and analysis were guided by the strict rules of the Dutch privacy laws and regulations. As the data in the study database could not be linked to individual patients, no informed consent or permission from a Medical Ethical Committee were necessary under Dutch and European laws.

### Measures

Segmentation of psychiatric care

Psychiatric care was divided into:

(1) outpatient psychiatric care (non-crisis outpatient care with or without BD medication);
  (1a) outpatient care with BD medication,
  (1b) outpatient care without BD medication,
  (1c) BD medication only, and
  (1d) no psychiatric care (neither outpatient care nor BD medication).

(2) crisis care (inpatient psychiatric care and/or intensive outpatient psychiatric crisis treatment).

Psychiatric care, in this study is specialized psychiatric care as delivered by psychiatrists or psychiatric providers. Medication recommended for BD is aimed at treating and/or preventing manic and depressive episodes and accompanying psychotic symptoms. This group of medications includes lithium, anticonvulsants, antipsychotics, and antidepressants.

#### Dependent variable: Discontinuity of outpatient psychiatric care

A patient experienced discontinuity of outpatient psychiatric care (discontinuity of care) when that patient did not receive outpatient psychiatric care in at least one of the quarters of 2016.

#### Independent variables: Age, sex, and urbanization

Age, sex, and urbanization were from 1-1-2015. The level of urbanization was based on the 4-digit postal code of the patient’s residence on 1-1-2015 (CBS, 2024).

#### Independent variables: Previous treatment in 2013 to 2014

The previous treatment per patient in 2013 to 2014 was measured in three ways:

*First*: The number of quarters for each of the four types of outpatient psychiatric care as defined previously was counted over the period 2013 to -2014.
*Second*: The amount of psychiatric treatment, using average national prices in euros, over 2013 to 2014 was determined:
(1) the amount of outpatient care measured as total costs of outpatient care;
(2) the amount of crisis care measured as total costs of crisis care.
*Third*: Alcohol and opioid dependence was determined using diagnoses of addiction (DSM-IV) or prescriptions with ATC codes for the treatment of alcohol and opioid dependence (N07BB and N07BC; Drugbank.ca, 2024). This was converted into two categories: any alcohol or opioid dependence or none. Information about other substance dependencies, like cannabis, was not available.

### Allocation of patients to psychiatric care providers

Patients were allocated in the dataset to the last psychiatric care provider from whom they had received outpatient psychiatric care in December 2014 to January 2015.

### Analysis

With forward stepwise logistic regression, predictive factors of discontinuity of care were identified. The binary variable discontinuity of outpatient psychiatric care (discontinuity of care) in 2016 was the dependent variable. Independent predictive variables were: age, sex, degree of urbanization, amount of outpatient care, amount of crisis care, number of quarters for each of the four types of outpatient psychiatric care, and alcohol or opioid dependence.

*First*, we inspected the univariate relationship between each of the potentially prognostic variables and discontinuity of care to verify the assumption of a linear relation of the continuous prognostic factors with discontinuity of care. The linearity assumption was checked with the log odds of the dependent variable discontinuity of care. Furthermore, collinearity between the prognostic variables was analyzed using Pearson correlation coefficients.

*Second*, forward stepwise logistic regression models were built. The threshold for adding a prognostic variable to the model was a significance level of.05. To decide if the model improved, the Akaike information criterion (AIC) was used (Yamashita et al., 2007). In the final model, the adjusted *R*^2^ (Nagelkerke *R*^2^) and the Hosmer and Lemeshow goodness-of-fit test were computed.

*Third*, practice variation between providers was determined. As a start, per provider, the expected number of patients with discontinuity of care was estimated from the final model of the stepwise logistic regression, thus adjusting for all relevant differences in available patient and care characteristics. Next, for each provider, the standardized event ratio (SER) was calculated as the ratio of the observed number of cases with discontinuity to the expected number of discontinuity (Woodward, 2004). The 95%-confidence interval was calculated using the method of Rothman Greenland (Soe & Sullivan, 2006). Testing the null hypotheses of no significant practice variation was done with a Chi-square test with k-1 degrees of freedom (k is the number of providers; Westerling, 1995).

All analyses were performed using SAS Enterprise guide 6.1 (SAS Institute Cary, NC, USA; see https://www.sas.com/en_us/home.html).

## Results

### Patient and care characteristics of the study sample

The study sample consisted of 2,355 patients. The average age was 48.9, women 49.1, men 48.7. Of all patients, 59.2% were women (Table 1). Many patients (43.1%) were living in more highly urbanized areas, much more than compared to all Dutch inhabitants (23%; CBS, 2024). Alcohol or opioid dependence was found in 5.6% of the patients.

**Table 1.** Patient and treatment characteristics of the study sample (*n* = 2,355).

Patient characteristics				Treatment characteristics			
	Category	*N*	%		Category	*n*	%
Sex	Female	1,394	59.2	Outpatient care^a^	<€3,000	650	27.6
					€3,000– €6,000	734	31.2
Age (years)	18–23	77	3.3		>€6,000	971	41.2
	24–33	244	10.4				
	34–42	367	15.6	Crisis care^b^	€0	1,719	73.0
	43–45	170	7.2		€0–€10,000	288	12.2
	46–60	1,010	42.9		€10,000–€70,000	307	13.0
	61–65	287	12.2		>€70,000	41	1.7
	66–69	200	8.5				
				Alcohol and opioid dependence			
Urbanization	High	1,013	43.1		Yes	133	5.6
	Medium-high	612	26.0				
	Medium	303	12.9	Continuous variables		*M* (*SD*)	
	Medium-low	247	10.5	Number of quarters^c^	Both O and M	5.97 (2.57)	
	Low	177	7.5		O only	0.90 (1.79)	
					M only	0.65 (1.39)	
					Neither O nor M	0.47 (1.29)	

a Amount of outpatient psychiatric care in the 2-year period.

b Amount of crisis care in the 2-year period.

c Number of quarters within the 2-year period given the types of care: O = Outpatient psychiatric care; M = Medication for bipolar disorder;

In each quarter of 2013 to 2014, a patient could receive any of the four types of outpatient psychiatric care. The patient could receive all quarters the same type of outpatient psychiatric care, but often the type of outpatient psychiatric care showed much variation. On average patients’ usage of outpatient care with BD medication was the highest (5.97 out of 8 quarters in 2013–2014), while patients’ average usage of neither type of outpatient psychiatric care was 0.47 out of 8 quarters (Table 1). A large group of 1,087 patients (46%) received outpatient care with BD medication during all eight quarters. The same treatment during all eight quarters was received by 57 patients (outpatient care without BD medication), 14 patients (only BD medication), and 16 patients (no treatment at all). The remaining 1,181 (50%) patients received all kinds of combinations of the four types of outpatient psychiatric care over the eight quarters.

### Linearity assumption of logistic regression

The univariate relationship between discontinuity and the continuous patient and care characteristics did not agree with the linearity assumption of logistic regression for age and amounts of outpatient and crisis care. Therefore, to match the nonlinearity in the relationship between these continuous prognostic variables and discontinuity, categorical scales were constructed (Table 1).

The age category 46 to 60 years contained most patients (42.9%) and the age category 18 to 23 years the least (3.3%, see Table 1). The amounts of outpatient and crisis care showed not only a nonlinear relation with discontinuity, but the distribution also shows spikes because of the structure of tariffs and very long thin tails. The amount of outpatient care was divided into three categories. The amount of crisis care was divided over four categories: 27% of patients received crisis care.

### The relationship between patient and care characteristics and discontinuity of care

Overall discontinuity of outpatient psychiatric care in 2016 was 12.1% (Table 2). The difference in the prevalence of discontinuity between men (13.6%) and women (11.1%) was not statistically significant (*p* = .067).

**Table 2.** The relationship between patient and care characteristics and discontinuity of care (*n* = 2,355).

Patient and care characteristics	Category discontinuity (%)		Category level	Variable level
			Odds ratio [95% CI]	Test results
Total sample		12.1%		
Patient characteristics				
Sex	Male	13.6%	Reference	χ^2^(1) = 3.37, *p* = .067
	Female	11.1%	0.79 [0.62, 1.02]	
Age (years)	18–23	31.2%	5.06 [2.97, 8.61]	χ^2^(6) = 64.14, *p* < .001
	24–33	21.3%	3.02 [2.07, 4.42]	
	34–42	10.9%	1.37 [0.92, 2.03]	
	43–45	15.3%	2.02 [1.26, 3.24]	
	46–60	8.2%	Reference	
	61–65	14.3%	1.86 [1.25, 2.78]	
	66–69	10.0%	1.24 [0.74, 2.07]	
Urbanization	High	13.9%	1.42 [0.94, 2.14]	χ^2^(4) = 10.54, *p* = .032
	Medium-high	10.6%	1.04 [0.66, 1.64]	
	Medium	10.2%	Reference	
	Medium-low	14.6%	1.50 [0.90, 2.50]	
	Low	7.3%	0.70 [0.35, 1.37]	
Psychiatric care characteristics				
Outpatient psychiatric care^a^	<€3,000	16.2%	Reference	χ^2^(2) = 16.80, *p* < .001
	€3,000–€6,000	12.3%	0.74 [0.54, 0.98]	
	>€6,000	9.4%	0.54 [0.40, 0.72]	
			
Crisis care^b^	€0	11.8%	Reference	χ^2^(3) = 11.52, *p* = .009
	€0–€10,000	12.2%	1.04 [0.71, 1,52]	
	€10,000–€70,000	12.1%	1.03 [0.71, 1.50]	
	>€70,000	29.3%	3.11 [1.56, 6.19]	
			
Alcohol opioid dependence	No	12.0%	Reference	χ^2^(1) = 0.61, *p* = .436
	Yes	14.3%	1.22 [0.74, 2.02]	
			
Number of quarters^c^	Both O and M		0.70 [0.67, 0.74]	*z* = −15.23, *p* < .001
	O only		1.47 [1.40, 1.55]	*z* = −14.11, *p* < .001
	M only		0.94 [0.85, 1.03]	*z* = −1.29, *p* = .1983
	Neither O nor M		1.47 [1.37, 1.57]	*z* = 10.61, *p* < .001

a Amount of outpatient psychiatric care in the 2-year period.

b Amount of crisis care in the 2-year period.

c Number of quarters within the 2-year period given the types of care: O = Outpatient psychiatric care; M = Medication for bipolar disorder;

Age and previous care in 2013 to 2014 (the amount of outpatient care, the amount of crisis care, and number of quarters) were related to discontinuity of outpatient psychiatric care in 2016 (Table 2). Discontinuity of care was most likely among the younger age groups of 18 to 23 years (31.2%) and 24 to 33 years (21.3%) and lowest in the age group of 46 to 60 years (8.2%). Previous outpatient care showed the highest discontinuity (16.2%) in those receiving relatively little outpatient care (costs of €3,000 or less), and the lowest (9.4%) in the few who received more than €6,000 of outpatient care. Previous crisis care showed, in 2016, the highest discontinuity (29.3%) in the small group receiving the most crisis care (costs of €70,000 or more). The lowest prevalence of discontinuity (11.8%) was found in the group without previous crisis care. Sex, urbanization, and alcohol and opioid dependency in 2013 to 2014 were not related to discontinuity of care in 2016 (Table 2).

Patients who had received outpatient care with BD medication during more quarters in 2013 to 2014 showed less discontinuity (odds ratio = 0.70, *p* < .001; Table 2). The risk of discontinuity was higher in patients with a history of receiving either outpatient care without BD medication (odds ratio = 1.47, *p* < .001), or more quarters without either outpatient care nor BD medication (odds ratio = 1.47, *p* < .001).

The large group of 1,087 patients who received outpatient care with bipolar disorder medication in all eight quarters of 2013 to 2014 showed a very low discontinuity (4%) in 2016. The small group of 57 patients receiving outpatient care without medication during the preceding eight quarters were at a much higher risk of discontinuity (53%).

### Logistic regression

The final logistic regression model included the proposed prognostic factors for discontinuity: age, amount of outpatient care, the number of quarters receiving outpatient care with BD medication, and the number of quarters receiving BD medication only (Table 3). Using other prognostic factors did not improve AIC. The adjusted *R*^2^ for the final model was 23.3%.

**Table 3.** Regression parameters of the final logistic regression model obtained from stepwise forward variable selection (*n* = 2,355).

Patient and care characteristics	Category	ln(OR)	*SE*	χ^2^	*p* value	OR 95% CI
Patient characteristics						
Age (years)	18–23	Reference				
	24–33	0.158	0.170	0.864	.353	1.17 [0.84, 1.63]
	34–42	−0.202	0.172	1.388	.239	0.82 [0,58, 1.14]
	43–45	0.210	0.214	0.965	.326	1.23 [0.81, 1.88]
	46–60	−0.396	0.131	9.088	.003	0.67 [0.52, 0.87]
	61–65	0.217	0.176	1.516	.218	1.24 [0.88, 1.76]
	66–69	−0.092	0.229	0.160	.689	0.91 [0.58, 1.43]
Psychiatric care characteristics						
Outpatient psychiatric care^a^	<€3,000	Reference				
	€3,000– €6,000	0.008	0.100	0.007	.935	1.01 [0.83, 1.23]
	>€6,000	−0.220	0.099	4.952	.026	0.80 [0.66, 0.97]
Number of quarters^b^	Both O and M	−0.366	0.025	220.807	*p* < .001	0.69 [0.66, 0.73]
	M only	−0.294	0.053	30.927	*p* < .001	0.75 [0.67, 0.83]
Intercept		0.217	0.140	2.397	.122	1.24 [0.94, 1.63]

a Amount of outpatient psychiatric care in the 2-year period.

b Number of quarters within the 2-year period receiving types of care: O = outpatient psychiatric care; M = medication for bipolar disorder.

### Practice variation

**Figure 2.**
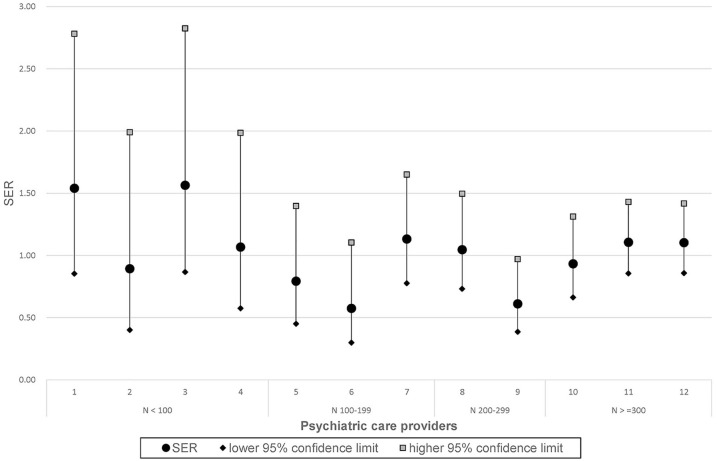
Standardized event ratios (SER) and 95% confidence intervals of 12 psychiatric care providers.

Per provider, the observed number of patients with discontinuity and the expected number of patients with discontinuity from the logistic regression model were obtained. Dividing the observed number by the expected number produced standardized event ratio (SER) per provider. The corresponding 95% confidence intervals were calculated. Figure 2 shows SER scores and the corresponding confidence intervals for all care providers. In accordance with expectations, the larger the number of patients, the narrower the confidence intervals became. A SER less than 1 means less observed cases than expected, a SER above 1 means there were more observed cases than expected. If the confidence interval does not overlap with 1, then the SER differs significantly from 1. Only one provider had 95% confidence intervals below 1, while none were above 1.

To determine if the providers showed practice variation, we tested the null hypothesis that all SERs are equal to 1 using the corresponding chi-square test. The null hypothesis could not be rejected (χ^2^(11) = 14.2, *p* = .22), indicating that no evidence for practice variation could be shown.

## Discussion

We aimed to study predictive factors for discontinuity of outpatient psychiatric care among Dutch patients with BD receiving outpatient psychiatric care. We used registry data from the largest Dutch health insurance company and included a large cohort of 2,355 patients.

In our cohort, containing patients with BD who were receiving outpatient psychiatric care around 1-1-2015, only 12.1% showed discontinuity of such care in 2016. This low level of discontinuity is reassuring and bears testimony to the efforts made by the national networks of BD care providers and patients working together to improve care (‘Kenniscentrum bipolaire stoornissen,’ 2024). Although we did expect to find quite good levels of continuity of care, this very high percentage is more than we had expected. Mental health in the Netherlands has to deal with severe shortages in personnel, financial cut backs, and closure of facilities. Over the years, waiting times for treatment have escalated. This has led to initiatives aiming to rationalize treatment intensity and duration, favoring shorter over longer treatments. In spite of these developments, consensus about the importance of continuous treatment for severe mental illness, such as bipolar disorder, has endured. In the Netherlands, there is a very active and relatively well-organized patient community and the collaboration with professional organizations and other stakeholders in the health care are relatively good. There is consensus that investing in continuity of care for bipolar disorder, focusing not only on short-term symptom reduction, but investing in relapse prevention and optimal well-being and functioning is worthwhile. This is borne out by the current findings. However, we did find some predictors of discontinuity of care. First of all, as may be expected, previous patterns of care use predicted discontinuity in 2016. Discontinuity was very low (4%) in patients with continuity of outpatient care in all quarters of the preceding years of 2013 to 2014. The risk of discontinuity of outpatient psychiatric care was higher in younger patients, and in patients with less outpatient psychiatric care, more crisis care, less outpatient care with BD medication, or more outpatient care without BD medication over 2013 to 2014. Age, amount of outpatient psychiatric care, and type of outpatient psychiatric care in 2013 to 2014 predicted discontinuity in the final model. Sex, urbanization, and alcohol or opioid dependency were not significantly related to discontinuity.

Some practice variation in discontinuity of care between psychiatric care providers was found, but after adjusting for all available prognostic patient and care characteristics, evidence for practice variation could not be determined.

Previous care consumption is an important prognostic factor of future discontinuity of outpatient psychiatric care. We propose that previous care consumption can be used to design strategies to increase continuity of outpatient psychiatric care and focus resources on those patients who are most at risk of dropping out.

### Strengths and limitations

Our research has the following strengths. Healthcare insurance registry data provide relevant and valid information about common practices in the treatment of patients. Dutch health insurance data is complete and of high quality and is relatively unbiased, and large sample sizes are possible. We could analyze the influence of practice variation on discontinuity of outpatient psychiatric care between providers since each provider had at least 50 patients at baseline. Therefore, they had enough experience treating patients with BD to provide appropriate continuous outpatient psychiatric care. We were able to adjust for patient characteristics and psychiatric care history of the previous 2 years.

However, there are several limitations of our study.

First, a naturalistic study design is open to bias due to the selection of patients or providers. Characteristics of Dutch patients who choose health insurance from Zilveren Kruis may differ from other ones. Research shows an uneven distribution over the health insurance companies of patients receiving specialized mental healthcare (PWC, 2019). Providers also differ in their share of patients in 1% of the highest costs, an indication of differences in severity. Providers with less than 50 patients diagnosed with BD were not included and may differ in discontinuity of outpatient psychiatric care because of a different subpopulation or being less experienced.

Second, other unknown factors than the patient and care characteristics available from the health insurance registry may have influenced discontinuity of outpatient psychiatric care. Even though research on healthcare insurance registry data has its strength, the records provide no detailed information on the clinical and environmental aspects of patients nor detailed information about the provided treatment. It is very likely that both clinical and environmental factors influence the effects on continuity of care. Because the adjusted *R*^2^ was not very high (23.3%), we cannot completely rule out practice variation between providers. Further research on registry data complemented with more detailed clinical and care characteristics in relation to discontinuity of psychiatric treatment is recommended.

Third, a study with a follow-up period of more than 1 year may reveal more details about the relationship between continuity of care and patients’ care characteristics or practice variation.

## Conclusions

Among a large (*n* = 2,355) cohort of patients with BD who were receiving outpatient psychiatric care at baseline, a low 12.1% experienced discontinuity of care over one follow-up year. The best predictor of continuous care was previous continuity of care: among patients with previous continuity of outpatient care, the risk of discontinuity was much lower at 4%. This suggests that investing in continuity of care is a highly valuable strategy in BD. We found no substantial practice variation in discontinuity of outpatient psychiatric care between providers, suggesting that efforts to reduce such variation over the past decades have been successful. Discontinuity of outpatient psychiatric care for patients with BD and also for patients with schizophrenia coincides with adverse outcomes, including increased use of psychiatric crisis care and hospitalization (van der Lee et al., 2016, 2019, 2021). We therefore suggest that previous care consumption patterns will be used more systematically to identify patients at risk for discontinuing treatment and develop strategies to improve continuity of care.

Our findings are encouraging, but continuity of care requires resources that are not available everywhere in the world. However, even in low-resource communities, aiming for continuity of such care as it is available in patients with bipolar disorder, is likely beneficial. This would again require consensus amongst all involved that such investment is worthwhile and here, also, knowledge about factors that facilitate or hinder continuity of care is important. Comparative work across different countries and cultures would be very welcome but has rarely been done.

## References

[bibr1-00207640241278291] Aldinger F. Schulze T. G. (2017). Environmental factors, life events, and trauma in the course of bipolar disorder. Psychiatry and Clinical Neurosciences, 71(1), 6–17. 10.1111/pcn.12433 27500795 PMC7167807

[bibr2-00207640241278291] Andrilla C. H. A. Patterson D. G. Garberson L. A. Coulthard C. Larson E. H. (2018). Geographic variation in the supply of selected behavioral health providers. American Journal of Preventive Medicine, 54(6), S199–S207. 10.1016/j.amepre.2018.01.004 29779543

[bibr3-00207640241278291] CBS. (2024). Bevolking naar achtergrondkenmerken, 2016. Retrieved March 24, 2024, from https://www.cbs.nl/nl-nl/maatwerk/2017/38/bevolking-naar-achtergrondkenmerken-2016

[bibr4-00207640241278291] Correll C. U. Detraux J. De Lepeleire J. De Hert M. (2015). Effects of antipsychotics, antidepressants and mood stabilizers on risk for physical diseases in people with schizophrenia, depression and bipolar disorder. World Psychiatry, 14(2), 119–136. 10.1002/wps.20204 26043321 PMC4471960

[bibr5-00207640241278291] Crawford M. J. de Jonge E. Freeman G. K. Weaver T. (2004). Providing continuity of care for people with severe mental illness. Social Psychiatry and Psychiatric Epidemiology, 39(4), 265–272. 10.1007/s00127-004-0732-x 15085327

[bibr6-00207640241278291] D’Avanzo B. Barbato A. Monzio Compagnoni M. Caggiu G. Allevi L. Carle F. D’avanzo B. Di Fiandra T. Ferrara L. Gaddini A. Leogrande M. Saponaro A. Scondotto S. Tozzi V. D. Carbone S. Corrao G. Italian Ministry of Health, QUADIM Project, & Italian Ministry of Health, Monitoring and Assessing Diagnostic-Therapeutic Paths. (2023). The quality of mental health care for people with bipolar disorders in the Italian mental health system: The QUADIM project. BMC Psychiatry, 23(1), e15. 10.1186/s12888-023-04921-7 PMC10261835 37312076

[bibr7-00207640241278291] de Ruiter K. G. C. de Groot M. ben Yerrou R . (2013). Kwaliteit en kosten van de geleverde zorg rond geestelijke gezondheidszorg. Retrieved March 24, 2024, from http://docplayer.nl/5652972-Kwaliteit-en-kosten-van-de-geleverde-zorg-rond-geestelijke-gezondheidszorg.html

[bibr8-00207640241278291] de Ruiter K. G. C. van Greuningen M. van Gerwen L . (2015). Praktijkvariatie in de GGZ. Vektis Zorgthermometer. Retrieved March 24, 2024, from https://www.vektis.nl/uploads/Publicaties/Zorgthermometer/ZorgthermometerPraktijkvariatieindeggz.pdf

[bibr9-00207640241278291] Dols A. Korten N. Comijs H. Schouws S. van Dijk M. Klumpers U. Beekman A. Kupka R. Stek M. (2018). The clinical course of late-life bipolar disorder, looking back and forward. Bipolar Disorders, 20(5), 459–469. 10.1111/bdi.12586 29227034

[bibr10-00207640241278291] Drugbank.ca. (2024). ATC classification. Retrieved March 24, 2024, from https://www.drugbank.ca/atc

[bibr11-00207640241278291] Edbrooke-Childs J. Boehnke J. R. Zamperoni V. Calderon A. Whale A. (2020). Service- and practitioner-level variation in non-consensual dropout from child mental health services. European Child and Adolescent Psychiatry, 29(7), 929–934. 10.1007/s00787-019-01405-6 31542793 PMC7321904

[bibr12-00207640241278291] Fischer E. P. McCarthy J. F. Ignacio R. V. Blow F. C. Barry K. L. Hudson T. J. Owen R. R. Jr. Valenstein M. (2008). Longitudinal patterns of health system retention among veterans with schizophrenia or bipolar disorder. Community Mental Health Journal, 44(5), 321–330. 10.1007/s10597-008-9133-z 18401711

[bibr13-00207640241278291] Golberstein E. Rhee T. G. McGuire T. G. (2015). Geographic variations in use of medicaid mental health services. Psychiatric Services, 66(5), 452–454. 10.1176/appi.ps.201400337 25726983

[bibr14-00207640241278291] Hoertel N. Limosin F. Leleu H. (2014). Poor longitudinal continuity of care is associated with an increased mortality rate among patients with mental disorders: Results from the French National Health Insurance Reimbursement Database. European Psychiatry, 29(6), 358–364. 10.1016/j.eurpsy.2013.12.001 24439514

[bibr15-00207640241278291] Kenniscentrum bipolaire stoornissen. (2024). Retrieved March 24, 2024, from https://www.kenbis.nl

[bibr16-00207640241278291] Kim G. Dautovich N. Ford K. L. Jimenez D. E. Cook B. Allman R. M. Parmelee P. (2017). Geographic variation in mental health care disparities among racially/ethnically diverse adults with psychiatric disorders. Social Psychiatry and Psychiatric Epidemiology, 52(8), 939–948. 10.1007/s00127-017-1401-1 28589236

[bibr17-00207640241278291] Kroon J. S. Wohlfarth T. D. Dieleman J. Sutterland A. L. Storosum J. G. Denys D. de Haan L. Sturkenboom M. C. (2013). Incidence rates and risk factors of bipolar disorder in the general population: A population-based cohort study. Bipolar Disorders, 15(3), 306–313. 10.1111/bdi.12058 23531096

[bibr18-00207640241278291] Kupka R. W. Goossens P. J. J. van Bendegem M. (2015). Multidisciplinaire richtlijn bipolaire stoornissen. De Tijdstroom.

[bibr19-00207640241278291] Laan W. Does Y. van der Sezgi B. Smeets H. Stolker J. Wit N. Heerdink E. (2010). Low treatment adherence with antipsychotics is associated with relapse in psychotic disorders within six months after discharge. Pharmacopsychiatry, 43(06), 221–224. 10.1055/s-0030-1254090 20503150

[bibr20-00207640241278291] Lublóy Á. Keresztúri J. L. Németh A. Mihalicza P . (2020). Exploring factors of diagnostic delay for patients with bipolar disorder: A population-based cohort study. BMC Psychiatry, 20(1), 75. 10.1186/s12888-020-2483-y 32075625 PMC7031950

[bibr21-00207640241278291] Nosé M. Barbui C. Tansella M. (2003). How often do patients with psychosis fail to adhere to treatment programmes? A systematic review. Psychological Medicine, 33(7), 1149–1160. 10.1017/S0033291703008328 14580069

[bibr22-00207640241278291] NZA. (2024). Overview of the Dutch healthcare authority. Retrieved March 24, 2024, from https://www.nza.nl/english

[bibr23-00207640241278291] O’Neill B. Kalia S. Aliarzadeh B. Moineddin R. Alan Fung W. L. Sullivan F. Maloul A. Bernard S. Greiver M. (2019). Agreement between primary care and hospital diagnosis of schizophrenia and bipolar disorder: A cross-sectional, observational study using record linkage. PLoS ONE, 14(1), 1–15. 10.1371/journal.pone.0210214 PMC6322753 30615653

[bibr24-00207640241278291] Park J. H. Fernando K. Park Y. H. Park E. O. (2024). Global perspectives on bipolar disorder treatment: In-depth comparative analysis of international guidelines for medication selection. BJPsych Open, 10(3), e75. 10.1192/bjo.2024.27 PMC11060076 38586960

[bibr25-00207640241278291] Peen J. Schoevers R. A. Beekman A. T. Dekker J. (2010). The current status of urban-rural differences in psychiatric disorders. Acta Psychiatrica Scandinavica, 121(2), 84–93. 10.1111/j.1600-0447.2009.01438.x 19624573

[bibr26-00207640241278291] Pennap D. Zito J. M. Santosh P. J. Tom S. E. Onukwugha E. Magder L. S. (2020). Continuity of care and mental health service use among medicaid-enrolled youths. Medical Care, 58(3), 199–207. 10.1097/MLR.0000000000001255 32106164

[bibr27-00207640241278291] Plans L. Barrot C. Nieto E. Rios J. Schulze T. G. Papiol S. Mitjans M. Vieta E. Benabarre A. (2019). Association between completed suicide and bipolar disorder: A systematic review of the literature. Journal of Affective Disorders, 242, 111–122. 10.1016/j.jad.2018.08.054 30173059

[bibr28-00207640241278291] PWC. (2019). WOR 958 Verbetering compensatie voor GGZ-cliënten met zeer hoge kosten GGZ. Retrieved March 24, 2024, from https://open-pilot.overheid.nl/Details/ronl-03e5eda8-c90e-4905-812a-3627e437d0a7/1?hit=1&text=verbetering+compensatie+voor+GGZ-cliënten+met+zeer+hoge+kosten+GGZ

[bibr29-00207640241278291] Salloum I. M. Brown E. S. (2017). Management of comorbid bipolar disorder and substance use disorders. The American Journal of Drug and Alcohol Abuse, 43(4), 366–376. 10.1080/00952990.2017.1292279 28301219

[bibr30-00207640241278291] Soe M. M. Sullivan K. M. (2006). Standardized mortality ratio and confidence interval. Retrieved March 24, 2024, from https://openepi.com/PDFDocs/SMRDoc.pdf

[bibr31-00207640241278291] Spijker A. T. Van Zaane J. Koenders M. A. Hoekstra R. Kupka R. W. (2019). Bipolaire stoornissen en alcoholafhankelijkheid. Praktische aanbevelingen voor behandeling op basis van een systematische literatuurstudie. Tijdschrift Voor Psychiatrie, 61(6), 372. 29436699

[bibr32-00207640241278291] Uher R. Pallaskorpi S. Suominen K. Mantere O. Pavlova B. Isometsä E. (2019). Clinical course predicts long-term outcomes in bipolar disorder. Psychological Medicine, 49(7), 1109–1117. 10.1017/S0033291718001678 29950190

[bibr33-00207640241278291] van der Lee A. P. M. de Haan L. Beekman A . (2016). Schizophrenia in the Netherlands: Continuity of care with better quality of care for less medical costs. PLOS ONE, 11(6), e0157150. 10.1371/journal.pone.0157150 PMC4898758 27275609

[bibr34-00207640241278291] van der Lee A. P. M. De Haan L. Beekman A. T . (2019). Rising co-payments coincide with unwanted effects on continuity of healthcare for patients with schizophrenia in the Netherlands. PLoS ONE, 14(9), 1–13. 10.1371/journal.pone.0222046 PMC6742391 31513629

[bibr35-00207640241278291] van der Lee A. P. M. Kupka R. de Haan L. Beekman A. T. F . (2021). Rising co-payments and continuity of healthcare for Dutch patients with bipolar disorder: Retrospective longitudinal cohort study. BJPsych Open, 7(5), e155. 10.1192/bjo.2021.994

[bibr36-00207640241278291] Vassos E. Agerbo E. Mors O. Bøcker Pedersen C. (2016). Urban-rural differences in incidence rates of psychiatric disorders in Denmark. British Journal of Psychiatry, 208(5), 435–440. 10.1192/bjp.bp.114.161091 26678865

[bibr37-00207640241278291] Watson P. Mehra K. Hawke L. D. Henderson J. (2019). Service provision for depressed children and youth: A survey of the scope and nature of services in Ontario. BMC Health Services Research, 19(1), 947. 10.1186/s12913-019-4784-8 31818284 PMC6902427

[bibr38-00207640241278291] Westerling R. (1995). Components of small area variation in death rates: A method applied to data from Sweden. Journal of Epidemiology and Community Health, 49(2), 214. 10.1136/jech.49.2.214 7798053 PMC1060110

[bibr39-00207640241278291] White J. Gutacker N. Jacobs R. Mason A. (2014). Hospital admissions for severe mental illness in England: Changes in equity of utilisation at the small area level between 2006 and 2010. Social Science and Medicine, 120, 243–251. 10.1016/j.socscimed.2014.09.036 25262312 PMC4225455

[bibr40-00207640241278291] Woodward M. (2004). Epidemiology – study design and data analysis, second edition (2nd ed.). Chapman & Hall/CRC.

[bibr41-00207640241278291] Yamashita T. Yamashita K. Kamimura R. (2007). A stepwise AIC method for variable selection in linear regression. Communications in Statistics - Theory and Methods, 36(13), 2395–2403. 10.1080/03610920701215639

[bibr42-00207640241278291] Zyto S. Jabben N. Schulte P. F. J. Regeer E. J. Goossens P. J. J. Kupka R. W. (2020). A multi-center naturalistic study of a newly designed 12-sessions group psychoeducation program for patients with bipolar disorder and their caregivers. International Journal of Bipolar Disorders, 8(1), 26. 10.1186/s40345-020-00190-5 32869118 PMC7459037

